# The contribution of synaptic location to inhibitory gain control in pyramidal cells

**DOI:** 10.1002/phy2.67

**Published:** 2013-09-23

**Authors:** Frederic Pouille, Oliver Watkinson, Massimo Scanziani, Andrew J Trevelyan

**Affiliations:** 1 Howard Hughes Medical Institute, University of California San DiegoLa Jolla, 92093-0634, California; 2 Department of Physiology and Biophysics, University of ColoradoDenver, Colorado; 3 Cardiology Department, Papworth HospitalPapworth Everard, CB23 3RE, Cambridge, U.K; 4 Institute of Neuroscience, Newcastle UniversityNewcastle upon Tyne, NE2 4HH, U.K

**Keywords:** Basket cell, cerebral cortex, gain control, inhibition, parvalbumin, pyramidal cell, somatostatin

## Abstract

The activity of pyramidal cells is controlled by two opposing forces: synaptic inhibition and synaptic excitation. Interestingly, these synaptic inputs are not distributed evenly across the dendritic trees of cortical pyramidal cells. Excitatory synapses are densely packed along only the more peripheral dendrites, but are absent from the proximal stems and the soma. In contrast, inhibitory synapses are located throughout the dendritic tree, the soma, and the axon initial segment. Thus both excitatory and inhibitory inputs exist on the peripheral dendritic tree, while the proximal segments only receive inhibition. The functional consequences of this uneven organization remain unclear. We used both optogenetics and dynamic patch clamp techniques to simulate excitatory synaptic conductances in pyramidal cells, and then assessed how their firing output is modulated by gamma-amino-butyric acid type A (GABA_A_) receptor activation at different regions of the somatodendritic axis. We report here that activation of GABA_A_ receptor on the same dendritic compartment as excitatory inputs causes a rightwards shift in the function relating firing rate to excitatory conductance (the input–output function). In contrast, GABA_A_ receptor activation proximal to the soma causes both a rightwards shift and also a reduction in the maximal firing rate. The experimental data are well reproduced in a simple, four compartmental model of a neuron with inhibition either on the same compartment, or proximal, to the excitatory drive.

## Introduction

Pyramidal cells are the principal excitatory neurons in the cortex. They receive excitatory inputs primarily from other pyramidal cells, and this is directed on to dendritic spines located throughout the distal dendritic tree. In contrast the most proximal dendritic compartments, within about 50 μm from the soma, and the soma itself, receive no excitatory inputs (Fairen et al. 1984; Megias et al. 2001). Inhibitory synapses, on the other hand, are found on all dendritic compartments, with a higher density on those compartments not targeted by excitatory synapses, including the proximal dendrites, the soma, and the axonal initial segment. Many studies have highlighted how the dendritic location of synapses determines how they summate, yet we still do not know the functional significance of this asymmetrical arrangement of synaptic drives in pyramidal cells.

The interactions between synaptic excitation and inhibition are generally discussed in terms of the neuronal input–output function, also referred to as “gain control.” A distinction is made between divisive gain changes (changes in the slope of the input–output curve) and subtractive changes (e.g., a rightwards shift, or offset, in the input–output curve – reviewed in Silver 2010). There are clear theoretical predictions about the effects of inhibition directed either on to the same dendritic branch or on more proximal branches (Jack et al. 1983; Trevelyan and Watkinson 2005). However, most experimental studies of neuronal gain control have been limited to simulating excitatory and inhibitory inputs into the same cellular compartment, at the soma (Chance et al. 2002; Mitchell and Silver 2003; Shu et al. 2003). Cerebellar granule cells have proved useful, on account of their extreme electrotonic compactness and the limited number of synaptic inputs they receive, permitting a reasonable simulation, from a single electrode, of the total synaptic drive onto these cells (Mitchell and Silver 2003; Rothman et al. 2009). These studies have shown how synaptic noise (Mitchell and Silver 2003; Shu et al. 2003) and short term synaptic depression (Rothman et al. 2009) both impact on the slope of the input–output functions, and other factors, such as the amplitude of afterhyperpolarization, are also open to neuromodulation. Thus there are multiple potential mechanisms of gain control in neurons. The electrotonically compact nature of cerebellar granule cells, however, makes them an imperfect model for cortical neurons, and so questions regarding the significance of inhibition at different locations in pyramidal cells remain to be resolved.

A major obstacle to understanding pyramidal cell synaptic drives has been the difficulty of specifying the location of excitatory or inhibitory inputs with any accuracy. Several recent studies have attempted to derive input–output functions using visual stimuli, while modulating inhibition by optogenetic control of different interneuronal populations (Atallah et al. 2012; Lee et al. 2012; Lovett-Barron et al. 2012; Wilson et al. 2012), but drew very different conclusions. The cause of these differences is difficult to discern, but may relate to problems defining the true location of the synaptic drives. In these experiments, there is a presumption that parvalbumin-expressing interneurons project to the somatic region while somatostatin-expressing neurons project more distally, but these are only relative and not absolute axonal projection patterns, and have not been characterized fully for the entire populations which are optogenetically activated in these experiments. Furthermore, little is known about the dendritic location of excitatory drives elicited by patterned visual stimuli. In short, the relative location of excitatory and inhibitory inputs in these studies is not as clearly defined as we would like.

We therefore took an experimental approach which allowed absolute control over the amplitude and location of both excitatory and inhibitory drives. We recorded the firing rate in individual pyramidal cells, while simulating excitatory conductance either optogenetically in that same cell, or by dynamic patch clamp, while applying the gamma-amino-butyric acid type A (GABA_A_) agonist, muscimol through a second patch pipette. We also focused on steady-state conditions, as the divisive effects of increasing membrane noise may obscure the predicted divisive effects of proximal inhibition. We were thus able to make a clear functional distinction between GABA_A_ activation onto the same dendritic compartment as the excitatory drive which produces a rightwards shift in the input–output function (“subtractive” gain control), versus that located proximal to the excitatory drive which causes both a rightwards shift and also a reduction in the maximal firing rate (“divisive” gain control). We show this effect in both hippocampal and neocortical pyramidal cells, and further illustrate these results with a simple conductance-based, multicompartment model.

## Methods

### Animal experiments

#### Acute slices preparation

Acute slices (400 μm) of hippocampus were prepared from 3- to 4-week-old Wistar rats and incubated for 45–60 min in an interface chamber at 35°C in artificial cerebrospinal fluid (ACSF) equilibrated with 95% O_2_ and 5% CO_2_, containing (in mmol/L): NaCl 119, KCl 2.5, NaHPO_4_ 1.3, MgCl_2_ 1.3, CaCl_2_ 2.5, NaHCO_3_ 26, and glucose 11. The slices were kept at room temperature for 1–6 h before being placed in a submerged chamber for recordings at 30–31°C.

Acute slices (400 μm) of somatosensory cortex were prepared at an angle which preserves thalamocortical connections (Agmon and Connors 1991), from 6- to 9-week-old mice expressing channelrhodopsin 2 (ChR2) in the large Layer 5 pyramidal cells. The slices were cut in a modified solution containing (in mmol/L) NaCl 83, KCl 2.5, MgSO_4_ 3.3, NaH_2_PO_4_ 1, glucose 22, sucrose 72, CaCl_2_ 0.5, and incubated in a submerged chamber at 34°C for 30–45 min, then at room temperature for 1–6 h in the same solution before being transferred to a recording chamber with normal ACSF maintained at 30–31°C.

#### Electrophysiology

Pyramidal neurons recorded in hippocampus (CA1 area) or in somatosensory cortex (layer 5b) were visually identified using infrared differential interference contrast videomicroscopy. The identity of ChR2-expressing layer 5b pyramidal cells was further confirmed based on their fluorescence and electrical response upon illumination at 470 nm. Whole cell recordings were performed with patch pipettes (soma: 4–6 MΩ; dendrite: 8–15 MΩ, 296–445 μm from the soma, 0–149 μm from the border with the lacunosum moleculare) containing (in mmol/L): potassium gluconate 150, 1.5 MgCl_2_ 1.5, HEPES 5, EGTA 1.1, and phosphocreatine 10 (pH = 7.25, 280–290 mOsm). Alexa 488 or 594 (5–10 μmol/L) was also included for neocortical pyramidal neurons to ensure that their primary dendrite was not severed. Series resistance was uncompensated but monitored continuously using negative voltage steps; recording sessions with series resistances larger than 15 MΩ (soma) or 60 MΩ (dendrite and dynamic clamp) were discontinued. Voltage measurements were not corrected for the experimentally determined junction potential (−11.7 ± 1.0 mV, *n* = 3) (Pouille et al. 2009). All experiments were performed in the presence of the GABA_B_ receptor antagonist CGP54626 (1–2 μmol/L), the 2-amino-3-(3-hydroxy-5-methyl-isoxazol-4-yl)propanoic acid (AMPA)/Kainate receptor antagonist 2,3-dihydroxy-6-nitro-7-sulfamoyl-benzo[f]quinoxaline-2,3-dione (NBQX, 25 μmol/L), and the N-methyl-D-aspartate (NMDA) receptor antagonist R-(–)-3-(2-carboxypiperazine-4-yl)-propyl-1-phosphonic acid (RS-CPP, 25–50 μmol/L).

#### Simulations of synaptic conductance: muscimol puff, dynamic clamp, and optogenetics

To activate GABA_A_ receptors on the membrane of a recorded CA1 pyramidal cell, the tip of a patch pipette (2–4 MΩ) filled with the GABA_A_ agonist, muscimol (Tocris Cookson, 10 μmol/L), was placed 18–95 μm from the cell membrane (its soma or the dendritic recording site), and puff series (1–60 puffs, 5–20 msec long puffs, 1.3–40 Hz) separated by 10 sec, were applied with a Picospritzer III (Parker Hannifin Corporation, Cleveland, OH). Artificial conductances were applied to the soma or the dendrites of pyramidal cells using a custom-made dynamic clamp interface as described previously (Pouille and Scanziani 2004). In short, the local voltage is measured in current clamp mode and the injected current is instantly calculated according to the following equation


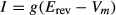
(1)

As the voltage is varying continuously, this results in a variable current injection. Excitatory conductances (*E*_rev_ = 0 mV) were simulated as a 1 sec, square-pulse function, starting 0.5 sec after the onset of the muscimol application. Inhibitory conductances (*E*_rev_ = −85 mV), also lasting 1 sec, were started 0.5 sec before ChR activation. ChR2-expressing layer 5b pyramidal cells were activated by a 1 sec squared light pulse delivered by a 5-W luxeon blue LED attached to the epifluorescence pathway of a Zeiss Axioscope II. The illumination beam was centered on the soma or on the apical dendrite (L2/3, 593–667 μm from the soma) of the recorded pyramidal cells. The excitatory conductance (*g*_E_) evoked by shining light at the soma or on the dendrite of the recorded cell was measured as the initial slope of the evoked current (ChR2PSC) divided by the driving force (when recording in voltage clamp). This method was chosen because large ChR2 currents triggered an action current that rendered measurement of the peak of the ChR2PSC impossible.

The frequency of action potentials (APs) was measured within the last 400 msec of the excitation injection period in both dynamic clamp/optogenetics. To determine the maximal action potential frequency (“plateau firing rate”) and the excitatory conductance at which half-maximal frequency is reached (“half max *G*_excit_”, σ), plots of AP frequency versus excitatory conductance were fitted with a sigmoidal function (sigmoid, three parameters, *F*_max_, *N*, and σ):

Firing frequency,


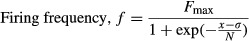
(2)

where *F*_max_ = plateau firing rate, *N* = slope, σ = half max *G*_excit_.

Dendritic membrane potential was measured immediately prior to the action potential. Data were recorded with Axopatch 200A, Axopatch 200B, or Multiclamp 700A amplifiers (Molecular Devices, Sunnydale, CA); acquisition (5–10 kHz digitization) and analysis were performed with pCLAMP 9.2 software (Molecular Devices) and Axograph X 1.3.5 (Axograph Scientific, Berkeley, CA). Average values are expressed as means ± SEM.

### Modeling

Modeling was performed to provide a simple, intuitive illustration of the phenomenon we describe in our experimental studies, showing the same inhibitory effects in a conductance-based model neuron. We simulated fluctuating synaptic drives in a simple, four compartment neuronal model, using the simulation package neuron (Hines and Carnevale 2001). The neuron comprised single axonal and somatic compartments, and a two compartment dendrite, with a shorter, thicker proximal dendritic compartment (L = 50 μm; diameter = 2 μm), and a long, thin, distal compartment (L = 500 μm; diameter = 0.5 μm). Active Hodgkin–Huxley type sodium and potassium conductances were located on the soma and axon, and action potential rates were recorded at the distal end of the axon. The dendrites contained passive and synaptic conductances. Resting membrane potential was −74 mV and input resistance was 350 MΩ (measured with sustained hyperpolarizing pulses delivered to the somatic compartment). Ten excitatory synaptic conductances (*E*_rev_ = 0 mV) were located on the distal compartment, equally spaced along its length (see schematics in Fig. 3). We examined two different patterns of inhibitory inputs (*E*_rev_ = −72 mV), arranging ten inhibitory synapses either on the same distal dendrite (“colocalized, distal inhibition”) or on the proximal dendrite (“proximal inhibition”). Both inhibition and excitation were applied as persistent conductances, albeit including a small amount of noise, modeled as a fluctuating conductance, with a coefficient of variance of 0.1.

## Results

### The relative location of inhibition and excitation dictates the type of gain control

**Figure 1:**
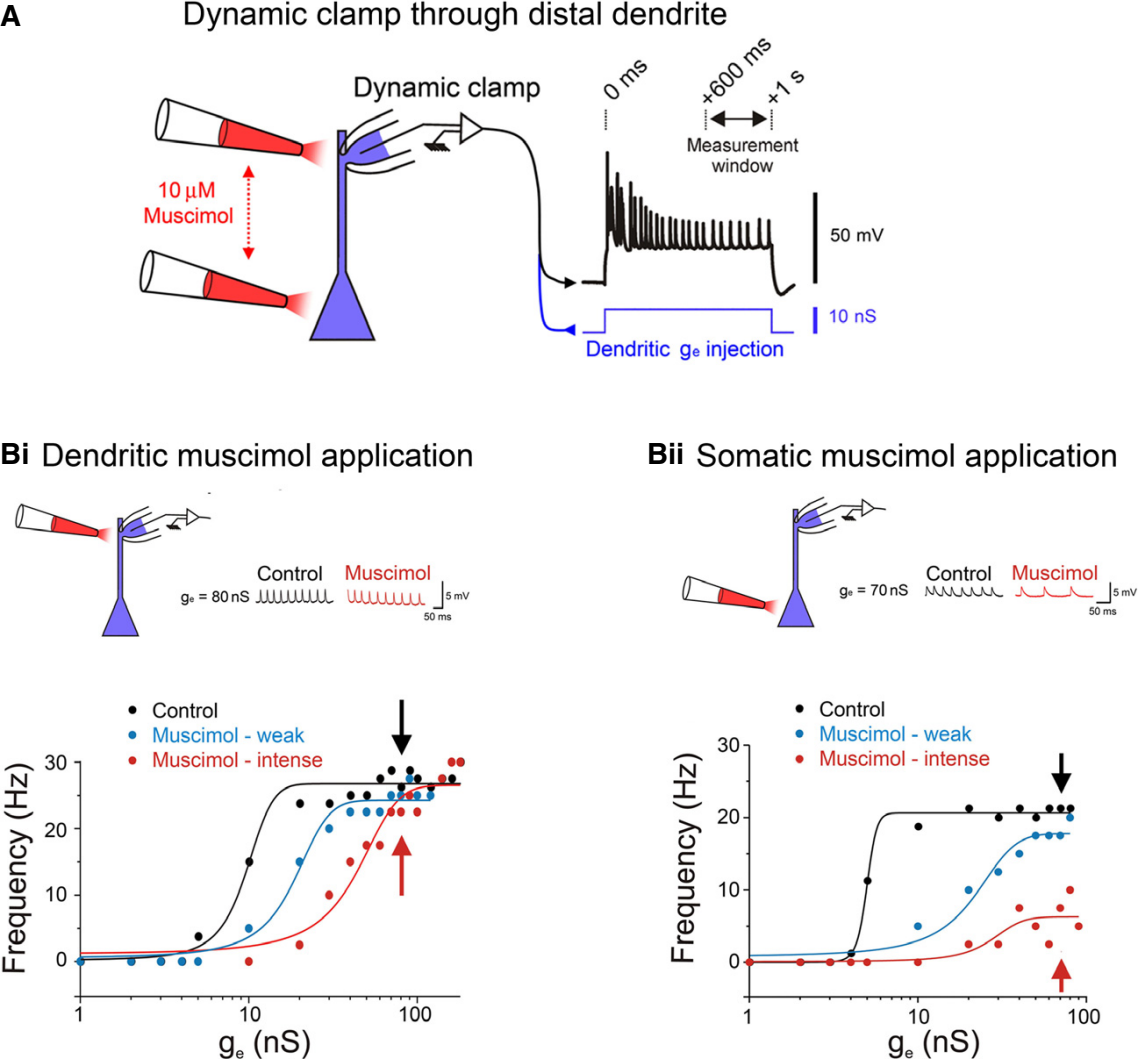
Example recordings showing a qualitatively different inhibitory effects arising in the distal versus the proximal dendrites. (A) Schematic showing the two different experimental protocols, with application of the GABA_A_ agonist, muscimol, either to the soma and proximal apical dendritic trunk, or to the more distal apical trunk close to the location of the patch pipette. A steady-state excitatory conductance (E_rev_ = 0 mV) was simulated by dynamic clamp over a 1 sec period, and the steady-state firing rate was derived from the final 400 ms of this. (B) Input–output IO functions at different levels of ambient muscimol for dendritic (Bi) and somatic (Bii) applications. Muscimol was applied by a continual series of low pressure puffs, and the concentration was varied by changing the frequency of pressure puffs. Note particularly, the different maximal firing rates in the two paradigms (arrows).

We took an experimental approach to investigate how inhibitory function varies across the dendritic tree of pyramidal cells. We initially studied rat CA1 pyramidal neurons, with all active conductances present and derived input–output functions for excitatory drive at appropriate sites on the dendritic tree. We patched on to the large apical dendritic trunk of CA1 pyramidal cells (296–445 μm from the soma, 0–149 μm from the border with the lacunosum moleculare) to simulate excitatory conductances (*E*_rev_ = 0 mV) in dynamic clamp recording mode. The simulated excitatory conductance, through the dendritic dynamic patch clamp, was varied to derive the relationship between the excitatory drive and the firing rate, the cell's input–output function (Fig. 1). In a few cells, we recorded APs using a second, somatically located patch electrode, but as this showed that APs, and thus the firing frequency, could be unambiguously recorded also through the dendritic patch (Fig. 1A), thereafter, this single dendritic electrode was used to provide both input and measure output of the recorded cells. Cells were routinely silent at rest, but started to fire above a threshold level of excitatory conductance, and further increases in excitatory drive induced an initial sharp increase in firing, which then plateaued (Fig. 1B-i, black). Input–output functions were fitted with a sigmoidal function with three parameters (asymptote, slope, and EC_50_ [the excitatory drive which induces 50% maximal firing rate]).

The GABA_A_ agonist, muscimol, was delivered (picospritzer controlled pressure puffs through a micropipette) either to the same dendritic compartment as the patch electrode, or to the proximal apical trunk and soma. Different levels of inhibition were provided either by altering the pressure or the frequency of pressure pulses. When inhibition was applied to the same compartment as excitation, the effect was simply to shift the input–output function to the right (Fig. 1B-i, blue and red traces). Notably though, it was still possible to drive the cell to reach the same maximal firing rate (Fig. 1B-i, inset and arrowed data points; baseline *F*_max_ = 25.8 ± 0.4 Hz; with dendritic inhibition, *F*_max_ = 25.0 ± 0.6 Hz; Students *t*-test, *t*_s_ = 1.12, not significant). In contrast, when inhibition was applied to the proximal dendritic tree, there was rightwards shift of the input–output function, and a marked drop in the plateau level (Fig. 1B-ii, baseline *F*_max_ = 26.2 ± 1.0 Hz; with dendritic inhibition, *F*_max_ = 15.6 ± 2.3 Hz; *t*_s_ = 4.45; *P* < 0.001).

**Figure 2:**
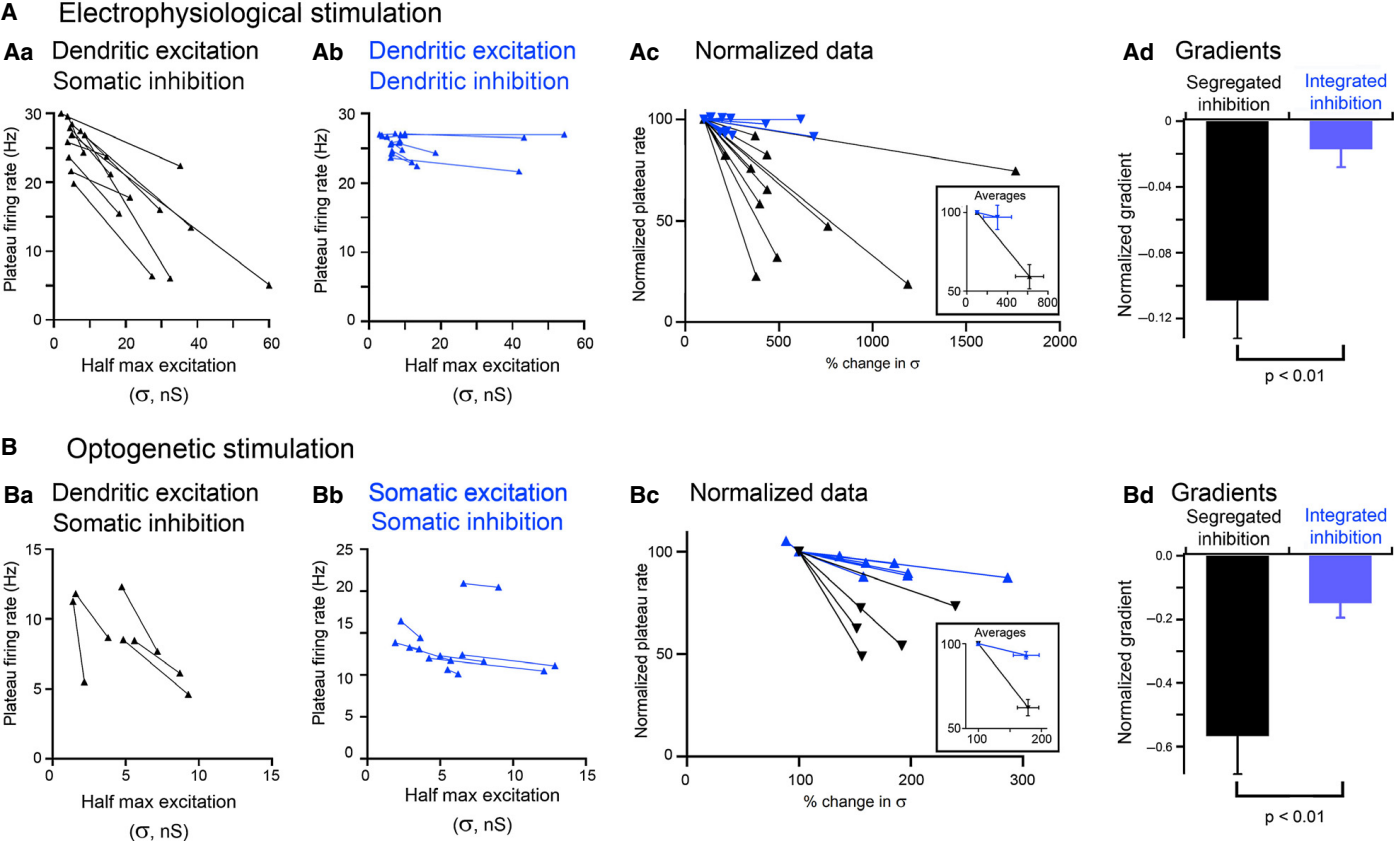
Pooled data showing a qualitatively different inhibitory effects arising in the distal versus the proximal dendrites. Pooled data show a significant, qualitative difference between the “colocalized” (inhibition and excitation on the same dendritic compartments) and “segregated” (inhibition proximal to excitation) patterns of inhibition. (A) Excitation provided by dynamic clamp delivered through a patch pipette. Muscimol applied to the proximal dendrites (Aa, “segregated inhibition”) causes both a rightwards shift and a great suppression of maximal firing, whereas muscimol applied to the same dendritic segment (Ab, “colocalized inhibition”) causes a simple rightwards shift of the IO function, with no reduction in maximal firing rate. (B) Equivalent data collected using optogenetic excitation. As in panel (A), proximally segregated inhibition causes a rightwards shift with suppression of maximal firing (Ba), whereas colocalized inhibition at the soma produces only a rightwards shift (Bb). This latter shows that it is the colocalized nature of inhibition rather than its specific location, which is the determinant of the rightwards shift in input–output IO function. There is a clear statistical difference between the colocalized and segregated patterns of synaptic arrangements (Ad, Bd).

In order to collate data from different cells, we fitted sigmoidal functions to the data, with three parameters, the maximal firing rate (*F*_max_), the slope (*N*), and half-maximal excitatory conductance (σ). The different effects of inhibition on to the same compartment, or on to more proximal regions of the dendritic tree, were well captured by simple plots of σ versus *F*_max_; Figure 2 shows the pooled data. Each paired data points (connected by lines), represents the *F*_max,_ σ parameters for input–output functions with and without inhibition. When muscimol was delivered to the same compartment as the dendritic patch electrode (Fig. 2A-b, blue), there were large changes in σ without affecting the maximal firing rate (*F*_max_), and so these lines connecting the data points are all horizontal. The normalized mean gradient (−0.017 ± 0.011; *n* = 11; Fig. 2A-d, blue) was not significantly different from zero, indicating a primarily subtractive effect. In contrast, proximally segregated inhibition increased σ and also reduced *F*_max_, to give negative slopes for the data pairs, indicating a divisive gain. Normalizing all these data to the baseline shows a clear separation of the data for the two conditions (Fig. 2A-c), with a highly significant difference in the gradients for the data pairs (Fig. 2A-d; proximally segregated inhibition gradient = −0.109 ± 0.023; *n* = 11; *t*_s_ = 3.65; *P* < 0.01). These experiments therefore show a clear qualitative difference between inhibition located on the distal and proximal branches of dendrites.

We next asked whether the same inhibitory effects could be seen also in neocortical pyramidal cells. Neocortical pyramidal cells present more difficulties regarding dendritic patching, so instead, we took an optogenetic approach, using mice which express ChR in subpopulations of neocortical pyramidal cells. In these experiments, we made somatic recordings from pyramidal cells expressing ChR, and directly illuminated focal spots in their dendritic tree, to activate conductances with similar reversal potential to glutamate receptors. This optogenetic protocol produced significantly more negative gradients than the equivalent paradigm using dynamic clamp (compare Fig. 2A and B; dendritic excitation and somatic inhibition: electrical, −0.109 ± 0.023; optogenetic, −0.566 ± 0.121; *t*_s_ = 5.34; *P* < 0.001), indicating a larger shunting effect in these experiments, but importantly the difference between proximal and dendritic inhibition was the same. Proximal inhibition again produced a divisive gain, reflected in the negative slope in the plots of EC_50_ versus *F*_max_ (−0.566 ± 0.121; *n* = 8).

We next asked whether the subtractive gain induced by dendritic targeting inhibition arose because the excitation and inhibition were colocalized to the same dendritic compartment, or if it were specific only to dendritic-targeted inhibition. We tested this by simulating excitation and inhibition both at the soma (Fig. 2B-b), thereby mimicking the colocalized condition for Figure 2A-b, but at a different location. As with the previous experiments, colocalizing inhibition with excitation produced a significantly reduced normalized gradient (proximally segregated inhibition, −0.566 ± 0.121 [*n* = 8]; colocalized inhibition, −0.148 ± 0.047 [*n* = 5]; *t*_s_ = 3.77; *P* < 0.01). This difference arises therefore from the colocalized nature of synaptic drives rather than from some unique feature of dendritic inhibition.

### The biophysical basis of the different inhibitory effects

**Figure 3:**
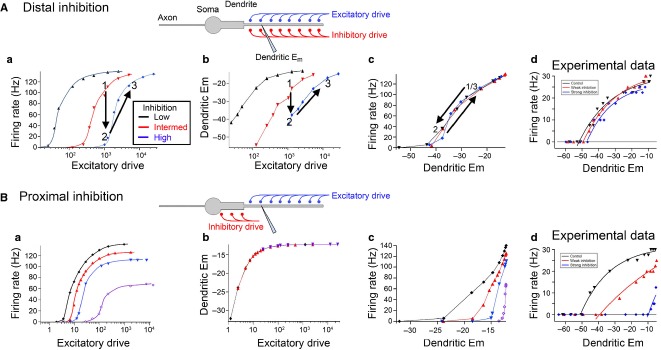
Neuronal simulations illustrate the differences between distal and proximal inhibition. The experimental data are reproduced easily in a simple four compartment model; schematics illustrate the two simulations which differ only in the location of the inhibitory synaptic drive. (A) Inhibition on to the same compartment as the excitatory drive causes a rightwards shift in the input–output function (Aa). The excitatory drive is calculated as the ratio of the synaptic conductance to the baseline passive conductance. Inhibitory conductance is about 200 times (black), 2000 times (red), and 10,000 times (blue) that of the passive baseline conductance. The rightwards shift in the input–output function is achieved by changing the local dendritic membrane potential (Ab), but importantly, inhibition colocalized with excitation does not change the relationship between the local dendritic membrane potential and the firing rate (Ac). (Ad) Experimental measures of the local dendritic membrane potential show little change in the relationship between dendritic E_m_ and firing rate despite the inhibition inducing a 115% change in the half-maximal excitatory drive (single exponential fits to the data with nonzero firing rates). (B) Proximal inhibition causes both a rightwards shift and a suppression of maximal firing rate, but does so without affecting the membrane potential at the site of the excitatory synapses (Bb), but rather by altering the transfer function (Bc) between the location of the excitatory drive and the action potential initiation site at the base of the axon. The black line represents the input–output function in the absence of shunting inhibition, and then with a proximal inhibition which is approximately 2000 times (red), 4000 times (blue), and 20,000 times (purple) the baseline passive conductance. (Bd) Experimental measures of the local dendritic membrane potential in one neuron, in the absence of muscimol (black), and for two different levels of proximal muscimol application (red and blue; single exponential fits to the data with nonzero firing rates).

The different inhibitory effects are best exemplified in a simple compartmental model of a neuron. As excitatory drive increases, the local dendritic membrane potential rises, but eventually, as the glutamatergic reversal potential is approached, further increases in excitatory conductance have less and less depolarizing effect (Fig. 3A-a and -b). Consequently, the firing rate starts to plateau. If inhibition is provided locally, there is an abrupt hyperpolarizing effect on the local dendritic potential (Fig. 3A; the shift from 1 to 2). We are now back on the steep slope of the sigmoidal curve, instead of at the plateau. By adding yet more glutamate conductance, the dendritic potential depolarizes to same level as before (Fig. 3A; the shift from 2 to 3), which then produces the same firing output. Notably, the relationship between the dendritic membrane potential at the site of the excitatory input, and the firing output does not change – all three plots exactly overlie each other (Fig. 3A-c), a feature which is also seen in the experimental data (Fig. 3A-d).

In contrast, when inhibition is added proximally to the glutamate conductance, the local dendritic depolarization provided by a given level of excitatory conductance does not change (Fig. 3B-b, all three plots overlie), and yet the firing output is dramatically reduced (Fig. 3B-a). When the local dendritic membrane potential at the location of the excitatory input is plotted against the firing rate, the plots are markedly altered by proximal inhibition (Fig. 3B-c). This is because proximal inhibition alters the electrotonic properties of the path between the excitatory synapse and the axon initial segment: the proximal dendritic tree becomes more leaky. Two features deserve mention. First, the threshold rises, as indicated by the rightwards shift of the point at which the plot deviates from 0 Hz. Second, the dynamic range of excitation is reduced, as indicated by the reduced range of dendritic membrane potentials lying between threshold and maximal firing. Once again, the model is well reproduced by experimental measures of the dendritic membrane potential in the presence or absence of proximal inhibition (Fig. 3B-d).

## Discussion

We have shown that the peculiar, asymmetric distribution of synaptic drives on to cortical pyramidal cells allows for two different “qualities” of inhibition, depending on the relative location of the excitatory and inhibitory drive. We have shown the same phenomenon in both hippocampal and neocortical pyramidal cells. These results are consistent with previous theoretical studies (Jack et al. 1983; Trevelyan and Watkinson 2005), experimental studies of the escape reflex in crayfish (Vu and Krasne 1992), and of inhibition in mouse visual cortex (Atallah et al. 2012; Wilson et al. 2012). These latter studies showed that the two different patterns of inhibition are provided by distinct populations of interneurons which target the peripheral dendrites or the proximal dendrites (and soma), respectively. We examined these issues at the subcellular level, to show that the different inhibitory effects reflect the relative distribution of excitatory and inhibitory inputs, rather than the specific location of the inhibitory inputs.

We analyzed the steady-state firing rates in pyramidal cells in order to address the location-specific inhibitory effects, independent of issues relating to the timing of inputs. These timing issues, however, are important considerations. The nonlinearity of action potential threshold means that random fluctuations of the membrane potential around threshold may trigger APs, even if the mean depolarization (or conductance level) remains below threshold. This noise-dependent effect has been suggested to underlie contrast invariance in visual cortex neurons (Anderson et al. 2000), and related phenomena where a sensory response is maintained in changed circumstances. These issues have also been studied using cerebellar granule cells, which are extremely electrotonically compact structures, and thus behave as if the inhibitory and excitatory inputs are on to the same compartment. Mitchell and Silver (2003) showed a similar effect to our colocalized inhibition and excitation (Fig. 2A-b and B-b) of inhibition with tonic conductances. But when they considered conductances which fluctuated because they arose from synaptic events happening at discrete time points, then the inhibitory effect also altered the slope as well as the offset of the input–output function (when plotted in terms of the frequency of inputs; see also Shu et al. 2003). They subsequently showed that short-term synaptic depression, by scaling down the excitatory drive, can be considered as a divisive gain shift, when the input–output function is plotted in terms of the input firing frequency, but not in terms of the conductance (Rothman et al. 2009). Divisive gain may also be manifested by altering the afterhyperpolarization, which may be regulated by various neuromodulators. Thus, there are multiple, parallel ways in which the input–output functions may be altered.

How do these different gain controls interact then? The tonic conditions, which we mapped out here, provide a good starting point, because they provide a close approximation of the instantaneous probability of firing, given a particular level of inhibitory and excitatory conductance. One can then consider how the instantaneous conductance varies. A particularly good example is provided by gamma rhythms, when basket cells provide a high variance pattern of inhibition. This is partly because individual synaptic connections provide large amplitude conductance, but with very fast kinetics, and this effect is further amplified by the coordination of different basket cells through their gap junction coupling. Another important feature is that the outputs of basket cells display very large synaptic depression. For both these reasons therefore, assessing the inhibitory effect of basket cells over a period of time may give a false impression of the power of these interneurons (Lovett-Barron et al. 2012). First, the high variance of the postsynaptic inhibition means that there exist windows of opportunity for pyramidal cells to fire between the inhibitory postsynaptic events. Second, their effect rapidly wains, and this is further complicated by the interplay between inhibitory populations (Lovett-Barron et al. 2012; Pfeffer et al. 2013) so that the somatic inhibition is subsumed by distal inhibition in sustained network activity (Pouille and Scanziani 2004).

The spike timing issues are pertinent to the results from optogenetic investigation of input–output functions (Atallah et al. 2012; Lee et al. 2012; Lovett-Barron et al. 2012; Wilson et al. 2012). Gap junction coupling between parvalbumin positive interneurons may create an oscillating inhibition from optogenetic activation of multiple interneurons. Thus, rather than providing a steady inhibitory effect, such synchronized spiking may still provide windows of opportunity for pyramidal firing between inhibitory postsynaptic potentials. Consequently, the main effect will be to alter the timing of APs (Cobb et al. 1995) and could give the appearance of only having a small effect on the total action potential count (Lovett-Barron et al. 2012). A further confound of the optogenetic experiments is that the measures are being made in oscillating networks, which means that the baseline activity and also the optogenetically stimulated activity of the interneurons is also likely to be oscillating, and the difference between the baseline and test states is highly unlikely to be the steady-state inhibitory effect we were able to study in our more straightforward assays. Finally, the onset of excitation can induce a supralinear dendritic excitation (Schiller et al. 2000; Lovett-Barron et al. 2012; Palmer et al. 2012). Such supralinear excitation was also evident in our own recordings as bursting at the start of the excitatory dynamic clamp, which arises from active NMDA and voltage-gated Ca^2+^ conductances in the dendrites (Lovett-Barron et al. 2012; Palmer et al. 2012). The implication of these studies is that for effective vetoing of pyramidal activity, as clearly happens at the areal boundaries of cortical seizures (Trevelyan et al. 2006; Schevon et al. 2012), inhibition is required to be directed to both locations of action potential generation: at the soma, where APs are the classical Hodgkin–Huxley, Na^+^-based variety (McCormick et al. 2007), and also in the dendrites where APs are sustained by slower kinetic currents (Schiller et al. 2000). These timing issues are beyond the scope of this paper, but they may yet be modeled by extrapolating from the steady-state effects we describe here.
